# The Optimal Weight Carriage System for Runners: Comparison Between Handheld Water Bottles, Waist Belts, and Backpacks

**DOI:** 10.3389/fphys.2020.571221

**Published:** 2020-09-30

**Authors:** Volker Scheer, Solveig Vieluf, Niko Bitter, Leon Christ, Hans-Christian Heitkamp

**Affiliations:** ^1^Health Science Department, Universidad a Distancia de Madrid (UDIMA), Madrid, Spain; ^2^Ultra Sports Science Foundation, Pierre Benite, France; ^3^Department of Exercise and Health, Institute of Sports Medicine, University of Paderborn, Paderborn, Germany

**Keywords:** running economy, energy cost of running, oxygen cost, loaded running, water bottle, waist belt, backpack

## Abstract

In endurance running, where fluid and nutritional support is not always readily available, the carriage of water and nutrition is essential. To compare the economy and physiological demands of different carriage systems, 12 recreational runners (mean age 22.8 ± 2.2 years, body mass index 24.5 ± 1.8 kg m^−2^, VO_2_max 50.4 ± 5.3 ml kg^−1^ min^−1^), completed four running tests, each of 60-min duration at individual running speeds (mean running speed 9.5 ± 1.1 km h^−1^) on a motorized treadmill, after an initial exercise test. Either no load was carried (control) or loads of 1.0 kg, in a handheld water bottle, waist belt, or backpack. Economy was assessed by means of energy cost (CR), oxygen cost (O_2_ cost), heart rate (HR), and rate of perceived exertion (RPE). CR [*F*(2,20) = 37.74, *p* < 0.01, η_p_^2^ = 0.79], O_2_ cost [*F*(2,20) = 37.98, *p* < 0.01, η_p_^2^ = 0.79], HR [*F*(2,18) = 165.62, *p* < 0.01, η_p_^2^ = 0.95], and RPE [*F*(2,18) = 165.62, *p* < 0.01, η_p_^2^ = 0.95] increased over time, but no significant differences were found between the systems. Carrying a handheld water bottle, waist belt, or backpack, weighing 1.0 kg, during a 60-min run exhibited similar physiological changes. Runners’ choice may be guided by personal preference in the absence of differences in economy (CR, O_2_ cost, HR, and RPE).

## Introduction

Running is a popular sport and interest and participation has grown over the last few decades (Ahmadyar et al., 2015; Scheer, 2019; Vitti et al., 2019), especially in trail, road, and ultra-endurance running (Scheer et al., 2020a). Maintaining appropriate fluid and nutritional intake is important to avoid decrements in performance and medical complications (Costa et al., 2016; Knechtle and Nikolaidis, 2018; Nikolaidis et al., 2018). Therefore, runners often carry nutrition and fluids during training runs and/or competitions, in different water carriage systems, such as handheld water bottles, waist belts, or backpacks (Scheer and Hoffman, 2018; Vincent et al., 2018). The weight of this additional load depends on distance, ambient temperatures, ease of access or availability of fluid/nutrition, personal choice, and the necessity of carrying additional equipment, such as mobile phones, keys, medical kit, and so on, and may easily add up to several kilograms, which is best carried in backpacks (Scheer et al., 2018a, 2020b). Keeping backpack loads to a minimum is recommended, as it is more economical with less physiological strain on the body (Scheer et al., 2018a), as well as using backpacks with back/front loading systems (Scheer et al., 2020b). However, for smaller and lighter loads or during shorter running distances, handheld water bottles or waist belts are often used (Vincent et al., 2018). These carriage systems are commonly reviewed in popular running magazines, but scientific data of the optimal choice of carrying extra weight in runners are still scarce, with the majority of studies pertaining to the military population, hikers, or walkers (Knapik et al., 1996; Simpson et al., 2017). Those studies generally examine loads that are heavier than those encountered in loaded running, as well as slower locomotion speeds, thus making the transfer of results to runners challenging or impossible (Knapik et al., 1996; Simpson et al., 2017). Studies on these populations showed that load position may influence energy expenditure (Lloyd and Cooke, 2000; Hudson et al., 2018), suggesting that load close to the center of mass may be the most effective way of carrying load, especially when walking (Knapik et al., 1996; Abe et al., 2004; Hudson et al., 2018).

For the assessment and comparison of load-carrying systems, the concept of running economy (RE) is often used, a multifactorial concept representing the combined function of the metabolic, cardiopulmonary, biomechanical, and neuromuscular system (Barnes and Kilding, 2015). RE can be expressed as oxygen consumption, oxygen cost to cover a given distance or energy cost (CR) (Fletcher et al., 2009; Shaw et al., 2014). For longer runs, the assessment of CR is of particular interest, as the energy yielded per liter of oxygen depends on the substrate that is metabolized, represented in the respiratory exchange ratio as an indicator of the mix of carbohydrate and fat utilization (Fletcher et al., 2009; Vernillo et al., 2015, 2017).

Of the few studies that have compared water carriage systems in runners (Scheer et al., 2018a, 2020b; Vincent et al., 2018), only one compared the economy of running with handheld water bottles and waist belts (Vincent et al., 2018). No changes in RE were observed; however, the testing period included only 5 min of loaded running, making comparisons for longer runs impossible (Vincent et al., 2018). Studies examining loaded backpack running with different weights and speeds demonstrated that with increasing backpack weight and speed RE deteriorated (Scheer et al., 2018a).

Energy expenditure can also be increased by biomechanical alterations such as postural sway and trunk movement produced by using backpacks (Folland et al., 2017; Hudson et al., 2018). Evenly distributing the weight in front/back backpack designs can be more economic during walking (Lloyd and Cooke, 2000) and running; however, this may only become apparent after prolonged running (Scheer et al., 2020b). Similarly, running with handheld water bottles can cause alterations of gait, trunk motion, and disruption of natural counterbalancing effects, possibly leading to increased energy demands (Vincent et al., 2018). However, no increase in energy demand was observed during a short 5-min testing period, possibly owing to energy-saving adjustments to preserve balance and gait, but whether these compensatory muscle actions can be maintained over longer running periods without an increase in energy expenditure is not known (Vincent et al., 2018).

Other measures of interest in the assessment of load-carrying systems in runners are cardiovascular effort and rates of perceived exertion (RPE), as with increasing load and running speed, heart rate (HR) and RPE increases (Scheer et al., 2018a, 2020b).

To our knowledge, no studies to date have examined the economy and physiological demands of handheld water bottles, waist belts, and backpacks during longer observational periods, to assess the optimal carriage system. Our aim was therefore to assess and compare the economy of handheld water bottles, waist belts, and backpacks in recreational runners, measuring the economy, cardiovascular effort, lactate, and RPE at individual submaximal running speeds during prolonged running periods of 60 min, with a weight of 1.0 kg. We hypothesized that economy would deteriorate with increasing running time among all carriage systems and that the waist belt and backpack would be more economical compared to the handheld water bottle due to the proximity of the load being close to the center of mass.

## Materials and Methods

Twelve recreational male runners participated in this explorative study (average age 22.8 ± 2.2 years, height 185.2 ± 6.2 cm, weight 84.2 ± 6.6 kg, body mass index 24.5 ± 1.8 kg m^−2^). Participants ran on average 3.2 ± 1.3 days per week and had a mean personal best 10-km time of 46 min 32 s ± 4 min 12 s. Runners were recruited through announcements at the local university and running clubs. Only healthy, male recreational runners, between the ages of 18 and 30 years, with a personal best 10-km time of less than 60 min, were allowed to participate. Female runners, or runners out with the inclusion criteria, were not allowed to participate. All participants were free of injury and any other concomitant health issues. Runners were instructed not to consume alcohol or caffeine before each test and to refrain from strenuous and exhaustive exercise at least 24 h prior to each test. Testing took place between October 2017 and January 2018, but each individual participant concluded testing within a 14-day period. All participants were informed of the study protocol prior to the first test and provided written, informed consent to participate. They received a medical check-up by the attending physician, consisting of history; examination of the cardiovascular, pulmonary, and musculoskeletal system; and resting electrocardiogram prior to the first test. They were familiar with running on a motorized treadmill. None of the runners were used to the carriage systems tested. Participants were instructed to wear the same clothes and footwear for all testing periods, as the type of footwear and shoe mass can influence the metabolic cost of running (Franz et al., 2012). The internal review board of the Ärztekammer Westfalen-Lippe and the Westfälische Wilhelms Universität, Münster, Germany (2017-084-f-S), approved all procedures.

### Handheld Water Bottle

**Figure 1:**
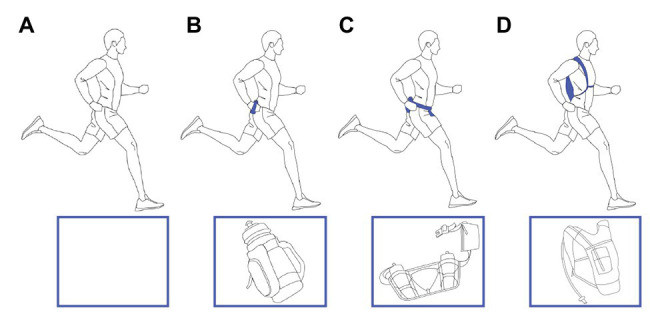
Illustration of the four different testing conditions, with background showing runner with the system and the front box the system only. From left to right, the figure shows runner **(A)** in control condition with no water carriage, **(B)** with handheld bottle, **(C)** with waist belt, and **(D)** with backpack.

The water bottle used was Ultimate Performance Kielder, net weight 114 g (Figure 1B). It contained a pouch for storage of other items and a hand strap for ease of holding the water bottle while running. The bottle itself had a capacity of just over 500 ml and when fully filled with fluid reached a total weight including the pouch of 685 g. Fluid consumption varies across individuals, running speed, sweat rates, body weight, prior hydration status, and ambient temperatures, and fluid replenishment should be guided by drinking to thirst; however, fluid replacement rates of 0.4–0.8 L h^−1^ have been suggested (Sawka et al., 2007; Scheer and Hoffman, 2018). Therefore, carrying approximately 500 ml of fluids during a 1-h run in recreational runners seems reasonable. Additionally, from our own empirical observations, runners often carry additional items such as a mobile phone, keys, and energy bars, totaling an additional weight of 315 g. These were fitted in the surrounding pockets of the pouch of the handheld water bottle, increasing the total weight to 1.0 kg, which was the testing weight for all conditions. The runners were allowed to hold the water bottle in the preferred hand and change it during the testing condition for personal comfort. They were not allowed to consume fluid from the testing device, to alter the testing weight, but were provided fluid by the researcher *ad libitum* throughout all the testing procedures.

### Waist Belt

The waist belt used was Lepfun E888, with two water bottles, net weight 121 g (Figure 1C). It contained a strap around the waist that could easily be adjusted for comfort, and runners were instructed to find the best fit for the testing period. The water bottles were filled with fluids, and the pockets of the waist belt contained additional weight to reach a total weight of 1.0 kg for all testing procedures.

### Backpack

The backpack used was Alando with 3-L water bladder, net weight 180 g (Figure 1D). It contained several straps for fitting comfortably around the trunk and reducing postural sway during running. Each runner was allowed to adjust the strapping to comfort. The total weight of the backpack was increased to 1.0 kg for all testing conditions and ease of comparison between the different carrying systems.

### Study Design

The study design included a total of five test days, at least 24 h apart. The first test included an incremental exercise test, followed by four tests in a simple randomized order, comparing the different carriage systems and control conditions as detailed below.

### Exercise Test

A graded incremental exercise test with lactate measurements and indirect calorimetry was performed on a motorized treadmill (h/p/cosmos Pulsar 3p, Traunstein, Germany), until exhaustion. The test started at 6.0 km h^−1^, increased by 2.0 km h^−1^ every 3 min with capillary blood lactate (bLA) measurements (analyzed with Biosem S-line EKF Diagnostics, Barleben, Germany) obtained from capillary blood from the right ear lobe at rest, after each increase in velocity and at the end of the test as described in detail elsewhere (Scheer et al., 2018b). From the lactate values measured, the lactate curve was obtained (Ergonizer, Software für sportmedizinische Leistungsdiagnostik, K. Röcker) for determination of individual running speed for the four experimental testing conditions (Scheer et al., 2019). Testing speed was set at 80% of individual anaerobic threshold (IAT) speed as has previously been used in loaded running studies (Scheer et al., 2020b). VO_2_max values were obtained in accordance to primary and secondary criteria as described in detail elsewhere (Scheer et al., 2018b). Velocity at VO_2_max (vVO_2_max) was obtained at the speed of VO_2_max in the incremental exercise test (Billat et al., 1995). Results of the initial exercise test and anthropometric data of participants can be seen in Table 1.

**Table 1:** Mean descriptive results of participants’ data and results from the incremental exercise test, with SD and 95% confidence interval (CI).

Variables	Mean	SD	Lower 95% CI	Upper 95% CI
Age (years)	22.8	2.2	21.4	24.2
Height (cm)	185.2	6.2	181.1	189.1
Weight (kg)	84.2	6.6	80.1	85.0
BMI (kg m^2^)	24.5	1.8	23.4	25.7
Max heart rate (bpm)	195.2	9.5	189.1	201.2
VO_2_max (ml kg^−1^ min^−1^)	50.4	5.3	47.1	53.8
Ventilation (L min^−1^)	160.2	28.0	142.3	178.0
RER	1.3	0.1	1.3	1.3
RR per min	58.8	9.2	53.8	64.7
vVO_2_max (km h^−1^)	16.3	0.9	15.7	16.9
Lactate at rest (mmol L^−1^)	0.8	0.2	0.6	0.9
Lactate end (mmol L^−1^)	11.0	1.4	10.1	11.9
RPE (Borg scale)	18.3	1.0	17.7	19.0
IAT lactate (mmol L^−1^)	3.3	0.4	3.0	3.50
IAT heart rate (bpm)	171.6	10.9	164.7	178.5
Speed IAT (km h^−1^)	11.8	1.3	11.0	12.7
Speed IAT 80% (km h^−1^)	9.5	1.1	8.8	10.1

BMI, body mass index; RER, respiratory exchange rate; RR, respiration rate; vVO_2_max, velocity at VO_2_max; RPE, rate of perceived exertion; IAT, individual anaerobic lactate threshold.

### Testing With Carriage Systems and Control

The following four tests were conducted in a randomized order, with each participant running for 60 min on the motorized treadmill at a speed of 80% IAT, either with no additional weight (control) or the different carriage systems each weighing 1.0 kg (handheld water bottle, waist belt, and backpack). See Figure 1 for an illustration of the carriage conditions. The following parameter were measured at time points 5, 30, and 60 min: ventilatory parameters, RER and oxygen uptake with breath-by-breath gas and volume analyzer (Metalyzer 3B, Cortex Biophysik, Leipzig, Germany), HR (Cardio 100 BT, Custo, Ottobrunn, Germany), RPE on the 6–20-point Borg scale (Borg, 1985), and bLA measurements. RE was calculated and expressed as the net O_2_ cost (ml kg^−1^ km^−1^) and net energy cost of running [(CR) kcal kg^−1^ km^−1^; (Fletcher et al., 2009; Scheer and Hoffman, 2018) during 5 min of testing (at time points 5, 30, and 60 min) at steady-state running conditions and filtered into 5-s blocks for data analyses. Net VO_2_ values were obtained from calculating the difference of VO_2_ at steady state minus VO_2_ at rest (standing in upright position) and for determination of the caloric equivalent of VO_2_ values were converted depending on RER values, which remained under 1.0 throughout all testing procedures (Péronnet and Massicotte, 1991; Scheer et al., 2018c).

### Statistical Analyses

We used SPSS (version 24.0; IBM Corp., Armonk, NY) for statistical analysis. Descriptive data were reported as means, SD, and 95% confidence intervals. Borg scale results, HR, CR, and O_2_ cost were analyzed with four carriage conditions (control, handheld bottle, waist belt, backpack) by three times (5, 30, and 60 min) repeated-measures analysis of variance (ANOVA). The level of significance was set at *p* < 0.05. Effect sizes are given as partial eta squares (η_p_^2^). Significant main effects and interaction effects were followed by Bonferroni-corrected pairwise comparison.

## Results

**Figure 2:**
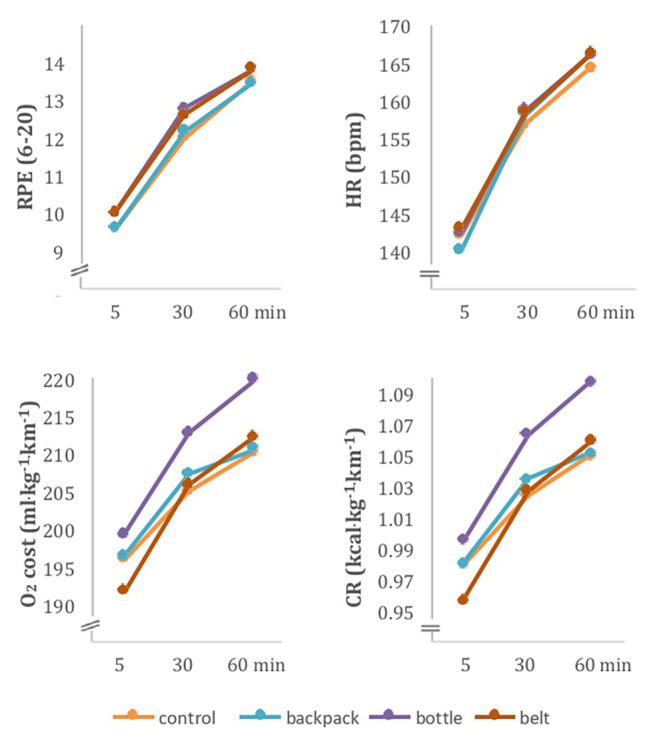
Means of RPE [rate of perceived exertion (Borg scale 6–20)], HR, O_2_ cost, and CR are illustrated per system [control, backpack, bottle (handheld water bottle), and belt (waist belt)] over time.

Participants’ data and results from the incremental exercise test are shown in Table 1. Descriptive statistics are shown in Table 2 and are graphically displayed in Figure 2. Individual data for CR is illustrated in Supplementary Material. RPE *via* the Borg scale increased over time [*F*(2,20) = 46.34, *p* < 0.01, η_p_^2^ = 0.82; all pairwise comparisons *p* < 0.01]. The main effect of systems [*F*(3,30) = 2.81, *p* = 0.058, η_p_^2^ = 0.22] and the time by systems interaction [*F*(6,60) = 0.70, *p* = 0.65, η_p_^2^ = 0.07] were not significant.

**Table 2:** Means and SD for different systems per measurement time for the group of runners.

Variables	System	5 min		30 min		60 min	
		Mean	SD	Mean	SD	Mean	SD
RPE (Borg scale)	Control	9.6	1.5	12.0	1.1	13.5	1.5
	Backpack	9.6	1.1	12.2	1.2	13.5	1.5
	Bottle	10.0	1.0	12.8	1.3	13.8	1.4
	Belt	10.0	1.7	12.6	1.1	13.8	0.8
HR (bpm)	Control	142.0	7.6	156.8	7.7	164.3	9.9
	Backpack	140.0	9.6	158.2	9.6	166.2	7.5
	Bottle	142.3	10.2	158.7	8.4	166.0	10.3
	Belt	142.8	8.2	158.4	7.0	166.1	10.5
Lactate (mmol L^−1^)	Control	1.4	0.5	1.2	0.4	1.3	0.4
	Backpack	1.6	0.5	1.3	0.3	1.3	0.3
	Bottle	1.4	0.3	1.2	0.5	1.3	0.3
	Belt	1.5	0.5	1.3	0.4	1.3	0.6
O_2_ cost (ml kg^−1^ km^−1^)	Control	195.9	31.0	205.0	24.45	210.1	31.6
	Backpack	196.3	32.4	207.3	20.2	210.6	22.5
	Bottle	199.3	35.2	212.7	30.4	219.7	27.3
	Belt	191.7	26.8	205.8	30.5	212.1	28.3
CR (kcal kg^−1^ km^−1^)	Control	0.98	0.15	1.02	0.12	1.05	0.16
	Backpack	0.98	0.16	1.04	0.10	1.05	0.11
	Bottle	1.00	0.18	1.06	0.15	1.10	0.14
	Belt	0.96	0.13	1.03	0.15	1.06	0.14

RPE, rate of perceived exertion; HR, heart rate; lactate; O_2_ cost, oxygen cost of running; CR, energy cost of running.

For HR, the ANOVA revealed a significant time by system interaction [*F*(6,54) = 4.15, *p* < 0.01, η_p_^2^ = 0.32). *Post hoc* comparisons were significant between all different time points for all systems (all *p* < 0.01), and at no time point the pairwise comparison for systems was significant. However, the interaction indicates that with time the differences between systems became smaller. Main effect of time [*F*(2,18) = 165.62, *p* < 0.01, η_p_^2^ = 0.95] was significant, whereas main effect of system [*F*(3,27) = 0.27, *p* = 0.09, η_p_^2^ = 0.03] was not.

Both CR and O_2_ cost increased with time [CR: *F*(2,20) = 37.74, *p* < 0.01, η_p_^2^ = 0.79; O_2_ cost: *F*(2,20) = 37.98, *p* < 0.01, η_p_^2^ = 0.79] and showed no significant differences between systems [CR: *F*(3,30) = 1.87, *p* = 0.16, η_p_^2^ = 0.16; O_2_ cost: *F*(3,30) = 1.94, *p* = 0.15, η_p_^2^ = 0.16]. Also, time by system interaction was not significant [CR: *F*(6,60) = 0.59, *p* = 0.74, η_p_^2^ = 0.06; O_2_ cost: *F*(6,60) = 0.51, *p* < 0.80, η_p_^2^ = 0.05].

bLA measurements were taken at the different time points (5, 30, and 60 min), to confirm that running speed was below IAT, at submaximal running speed. This was confirmed for all participants during all time points. In line with other ANOVA models, lactate values were compared for time points 5, 30, and 60 min. bLA increased over time [*F*(2,20) = 6.62, *p* < 0.01, η_p_^2^ = 0.95]. Also, main effect of system [*F*(3,30) = 0.96, *p* = 0.41, η_p_^2^ = 0.09] and time × system interaction [*F*(6,60) = 0.35, *p* = 0.90, η_p_^2^ = 0.03] were not significant.

## Discussion

This is the first study to examine the impact of three weight carriage systems (handheld water bottle, waist belt, and backpack) compared to no water carriage (control condition), in recreational runners, during a 60-min testing period, at individual submaximal running speeds on a motorized treadmill. Our aim was to compare the impact of the carriage condition on RE, cardiovascular effort, lactate, and RPE, and we hypothesized that economy would deteriorate with increasing running time among all carriage systems and that the waist belt and backpack would be more economic compared to the handheld water bottle due to the proximity of the load being close to the center of mass.

Our results showed that economy deteriorated over time across all systems; however, no one single system was more economic, suggesting that during a 60-min run, at submaximal running speed, with a load of 1.0 kg the choice of carriage system has no significant influence on economy, HR, lactate, or RPE.

Our hypothesis that the handheld water bottle would be less economic could not be confirmed. Carrying unilateral handheld loads up to 4 kg during walking can restrict upper limb biomechanics and result in a shifting of the center of mass (Birrell et al., 2007). If this applies to smaller loads, and especially during running, is less clear (Vincent et al., 2018). Comparing RE of a full (454 g) with a half-full (227 g) handheld water bottle and a waist belt (676 g) yielded no significant difference in RE, although running with a handheld water bottle altered gate and kinematics of running motion; however, this did not affect RE (Vincent et al., 2018). The authors speculated that because of energy-saving adjustments, balance and gait in the handheld water bottle were preserved during the 5-min running test, thus not altering RE; however, the authors recommended examining longer running durations, which may reveal additional differences and increase in energy expenditure over time (Vincent et al., 2018). The load we examined (1.0 kg) was heavier, and the testing period longer (60 min), and although CR and O_2_ cost were higher in the handheld water bottle condition compared to the waist belt and backpack, there were no statistical differences in economy between the systems. It is possible that, with a larger cohort, differences in RE may be more pronounced, and this will be worth exploring in future studies. We would also like to point out that in sports sometimes small, nonsignificant changes can be meaningful, especially for the individual, and that interindividual differences might contribute to nonsignificant results, as well as the order in which systems are tested, especially in small sample sizes. The observed differences in RE may be of interest for the individual runner, especially because RE can be improved with concurrent strength and endurance training, and even small changes of approximately 2–5% can lead to improvements in running performance (González-Alonso and Calbet, 2003; Denadai et al., 2017). We did not examine training interventions or adaptive responses to the carriage systems, but it is likely that training and habituation to one specific loading system may improve RE and possibly performance, but this will require further investigation. Similarly, it may be possible that the higher but nonsignificant difference in energy demand observed in carrying a handheld water bottle may provide sufficient practical differences to be reflected in performance times in a time trial or competition, compared to the waist belt or backpack; however, this will also require further investigation. It is also important to note that other parameters apart from RE are valuable in the assessment of economy in loaded running (Scheer et al., 2018a, 2020b), such as cardiovascular effort (HR) and RPE. Both HR and RPE did not differ significantly between loading systems in our cohort, suggesting that based on our data neither the handheld water bottle, waist belt, nor backpack had a superior economy on a group level during a 60-min run at constant speed on the treadmill.

We did not measure kinematic or gait parameters; however, even if running with the handheld water bottle altered biomechanics, it was not sufficient to alter RE to produce a significant difference between the systems. Whether there is indeed an energy-saving adaptive response with compensatory muscle actions, even in prolonged running, is currently unclear (Vincent et al., 2018).

In loaded backpack running, RE deteriorates with increasing weight, running time, and speed (Scheer et al., 2018a, 2020b). Load position may be important and can influence energy expenditure (Lloyd and Cooke, 2000; Hudson et al., 2018), suggesting that load close to the center of mass may be the most effective way of carrying load, especially when walking and carrying heavy loads (Knapik et al., 1996; Abe et al., 2004; Hudson et al., 2018). In loaded running, it has been shown that a front/back backpack design is more economic, especially after prolonged running (Scheer et al., 2020b). Postural sway and trunk movement from backpack usage may also negatively affect energy expenditure (Folland et al., 2017; Hudson et al., 2018).

Another important factor in assessing economy is the demand on the cardiovascular system, e.g., expressed by an increasing HR. This was documented in loaded backpack running, where HR increases over time, with running speed and backpack weight (Scheer et al., 2018a, 2020b), as well as in loaded walking (Macadam et al., 2017). This is in line with our own findings that HR increased significantly over time. No significant differences were found in cardiovascular effort between running with a full water bottle, a half-full water bottle, and a waist belt (Vincent et al., 2018). Our results confirm this, as we could not find significant differences in HR between the systems, albeit during a longer running period and with heavier weights.

Subjective perceptual methods, such as providing feedback to semistructured open-ended questions about the choice of carriage system immediately after the testing period or recording RPE measurements, may be useful in providing additional information, especially when physiological differences are small (Legg et al., 2003). We therefore, measured RPE at different time points and across the different carriage conditions; however, no significant differences were observed, suggesting that even subjectively there was no difference. Again, this is an interesting finding and would suggest that the selection of the preferred carriage system comes down to personal choice, in the absence of statistical differences in physiological measures or subjective assessments. Future studies should compare if personal preference matches individual data on economy and if economy changes during longer distances and longer testing duration at various speeds (fixed vs. self-selected) and slopes (uphill and downhill conditions) or in the field. Further testing a range of different weights and across sexes, as well as athletes from different athletic abilities with a larger sample, may provide useful information.

### Strength

The main strength of the study is that it compared the economy (CR, O_2_ cost, HR, and RPE) of three popular carriage systems (handheld water bottle, waist belt, and backpack) during prolonged running (60 min). This is the first study to examine this and describe the impact of carriage systems on economy during prolonged running. Previous studies examined economy during shorter periods, but transferring those results for prolonged running is not feasible. We also included a preceding exercise test, to determine the individual running speed for testing, so that all participants exercised at the same relative submaximal intensity. However, we recognize that there are still some limitations and weaknesses to our study.

### Limitations

Biomechanical aspects are important in assessing loaded running, but we were unable to perform this in the current study because of limited laboratory facilities; however, because we did not report significant differences in RE, significant biomechanical changes are unlikely, as they would have been measured by an increase in CR and O_2_ cost, but we recognize that this may have provided us with additional information. Another limitation of the study is the comparably small sample size. The testing weight was 1.0 kg for equal comparison between the load-carrying systems, but an examination of a range of different weights, testing speeds, and slopes may provide additional useful information. Additionally, fluids will be consumed while running, thus reducing the load to be carried, hence a further reason to test varying weights over different time periods, especially in running periods over 1 h, where these devices are mainly being used.

### Practical Applications

Our results suggest that carrying a handheld water bottle, waist belt, or backpack, weighing 1.0 kg, during a 60-min run exhibited similar physiological changes and that there were no significant differences in economy (CR, O_2_ cost, HR, and RPE). From a practical perspective, these results are important, considering the popularity of running and the need of carrying adequate nutrition and fluids in prolonged running, where fluid and nutritional support is not always readily available. Therefore, it is prudent to suggest that the individual runner may be guided by personal preference in selecting the carriage system of choice, in the absence of differences in economy.

## Conclusion

Carrying a handheld water bottle, waist belt, or backpack, weighing up to 1.0 kg, during prolonged runs of up to 1 h in recreational runners exhibited similar physiological changes, and there was no significant difference in the systems examined. We recommend that in the absence of significant differences in economy the runner selects the carriage system based on personal preference. Larger studies and assessing the impact of carriage systems on performance are also recommended.

## Data Availability Statement

The raw data supporting the conclusions of this article will be made available by the authors, without undue reservation.

## Ethics Statement

The studies involving human participants were reviewed and approved by The internal review board of the Ärztekammer Westfalen-Lippe and the Westfälische Wilhelms Universität, Münster, Germany (2017-084-f-S) approved all procedures. The patients/participants provided their written informed consent to participate in this study.

## Author Contributions

VS and H-CH contributed substantially to the conception and design of this study. VS, NB, and LC contributed to data collection. SV carried out the data analysis and interpretation together with VS, NB, LC, and H-CH. VS wrote the first draft of the manuscript, and all authors were involved in revising it critically. All authors gave final approval of the version to be published and agreed to be accountable for all aspects of this work.

### Conflict of Interest

The authors declare that the research was conducted in the absence of any commercial or financial relationships that could be construed as a potential conflict of interest.

## Supplementary Material

Supplementary Figure 1 Illustration of CR values after 5, 30, and 60 min of running with different carriage systems for each individual runner (1–12).
