# Supplementary figures and images for: The Optimal Weight Carriage System for Runners: Comparison Between Handheld Water Bottles, Waist Belts, and Backpacks
Source: Front Physiol. 2020 Sep 30;11:571221. doi: 10.3389/fphys.2020.571221 (PMC7561373; doi:10.3389/fphys.2020.571221)

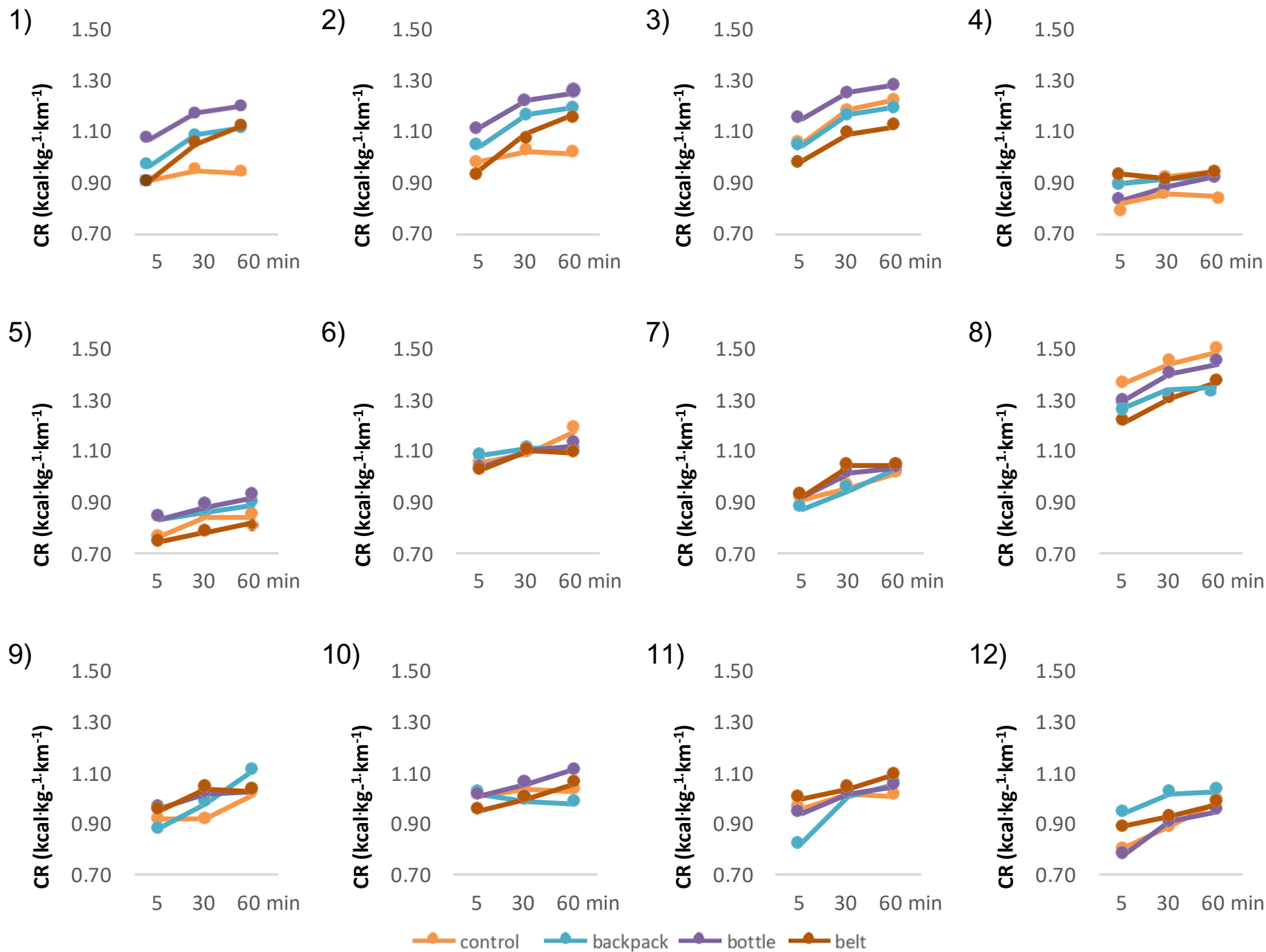

Supplement: Supplementary Figure 1 — Illustration of CR values after 5, 30, and 60 min of running with different carriage systems for each individual runner (1–12). [file Data_Sheet_1.pdf]
